# Thermodynamics of continental deformation

**DOI:** 10.1038/s41598-023-47054-3

**Published:** 2023-11-14

**Authors:** Ajay Kumar, Mauro Cacace, Magdalena Scheck-Wenderoth

**Affiliations:** 1 GFZ German Research Centre for Geosciences, Potsdam, Germany; 2 Faculty of Georesources and Materials Engineering, RWTH Aachen University, Aachen, Germany

**Keywords:** Geodynamics, Geophysics, Tectonics

## Abstract

Continental deformation is known to be controlled by the interplay between tectonic and gravitational forces modulated by thermal relaxation-controlled lithospheric strength leading to oscillations around an equilibrium state, or to runaway extension. Using data-driven thermomechanical modelling of the Alpine Himalayan Collision Zone, we demonstrate how deviations from an equilibrium between mantle dynamics, plate-boundary forces, and the thermochemical configuration of the lithosphere control continental deformation. We quantify such balance between the internal energy of the plate and tectonic forces in terms of a critical crustal thickness, that match the global average of present-day continental crust. It follows that thicker intraplate domains than the critical crust (orogens) must undergo weakening due to their increased internal energy, and, in doing so, they dissipate the acquired energy within a diffused zone of deformation, unlike the localized deformation seen along plate boundaries. This evolution is controlled by a dissipative thermodynamic feedback loop between thermal and mechanical relaxation of the driving energy in the orogenic lithosphere. Exponentially growing energy states, leading to runaway extension are efficiently dampened by enhanced dissipation from radioactive heat sources. This ultimately drives orogens with their thickened radiogenic crust towards a final equilibrium state. Our results suggest a genetic link between the thermochemical state of the crust and the tectonic evolution of silicate Earth-like planets.

## Introduction

**Figure 1:**
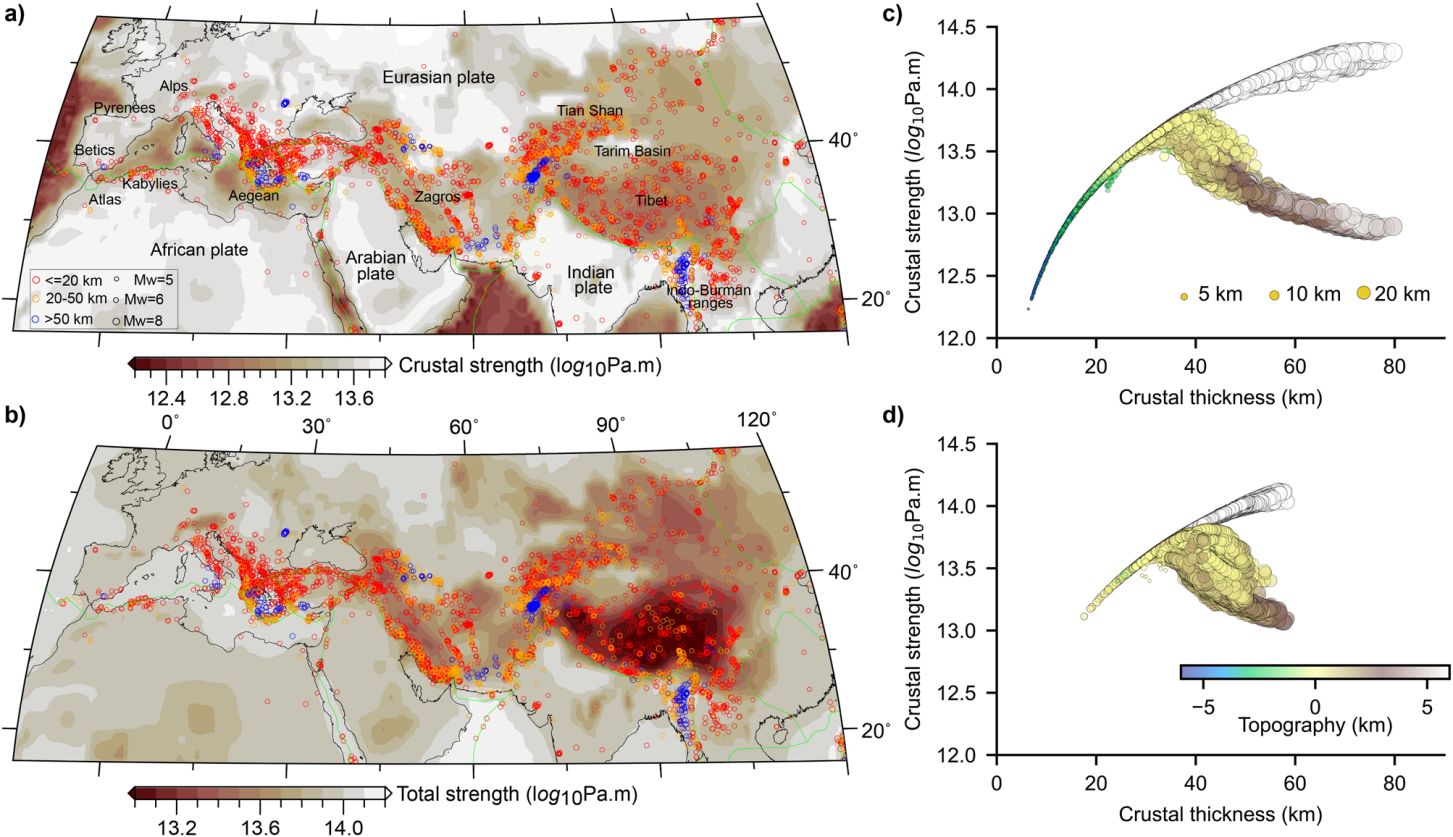
Long-term strength of the AHCZ. (**a**) Integrated strength of the crust and (**b**) of the lithosphere. Thermophysical and rheological parameters for the different geological layers are listed in Table 1 (see also “Methods”). Plate boundaries^76^ are plotted by green curves and earthquakes from ISC-GEM^77^ version 9 are overlaid by open circles, scaled by their magnitudes and colour-coded by their hypocentre depth (see legend in a). (**c**) Evolution of the integrated crustal strength as a function of its thickness^29^ for Mesozoic-Cenozoic tectonothermal ages^54^, including oceanic regions. In (**d**) the crust with tectonothermal ages older than Mesozoic-Cenozoic is plotted. The size of the circles is scaled by the thickness of the upper crust^29^ (see legend in **c**) and colour-coded by the observed topography^53^ (see legend in **d**). In the background, white circles show the evolution of crustal strength vs thickness corresponding to a model where heat production in the crust is neglected (see Supplementary Fig. S2 online). Maps were made using Generic Mapping Tools Version 6^78^.

Rigid plates moving relative to each other, an approximation of the outer shell of the Earth, has been successful in explaining the evolution of the oceanic lithosphere and laid the foundations to the kinematic theory of plate tectonics^1–3^. When applied to continents, plate tectonics fail to explain the observed complex deformation at collision zones and their interiors. An example of this complexity is the Alpine Himalayan Collision Zone (AHCZ), Fig. 1a. The AHCZ is the result of the closure of the paleo-Tethys ocean between the southern margin of Eurasia and the northern margin of paleo-continent Gondwana, and encompasses continental lithosphere of different tectonothermal ages (see Supplementary Fig. S1 online). Plate-wide compression, ongoing since Phanerozoic times, has produced a high degree of crustal differentiation (see Supplementary Fig. S1 online), and a rather diffused deformation as evidenced by the widespread distribution of earthquakes (Fig. 1a).

On geological time scales, the deformation of the continental lithosphere is explained by a description of the plate deforming as a viscous fluid^4,5^. Orogens that deform in response to an applied convergence are considered to be mechanically weaker than their foreland lithosphere^6^, a feature confirmed by data-driven lithospheric scale models of the Alps and Andes^7,8^. The physics driving this weakening has been ultimately related to mantle processes occurring during plate-plate collision (e.g. lithospheric-drip, delamination, or slab break-off), bringing hotter asthenosphere to shallow depths, thereby weakening the overlying orogenic lithosphere^9–11^. Time scales to relax the heat anomaly from such upwelling vary in a range from 8 Myrs up to 22 Myrs^12^. This entails that their influence is likely to be diffused within only a few 10’s Myrs. Previous studies indicate that orogens in the AHCZ are characterized by a deep thermal lithosphere-asthenosphere boundary which is indicative of a thick lithosphere likely limiting the heating of the orogenic lithosphere^13–16^ (see Supplementary Fig. S1 online). In addition, estimates of seismogenic strain rates in intraplate settings are indicative of a stress state that varies on time-scales around 100’s Myrs, which are therefore typical for plate-boundary reorganization^17,18^. These observations strongly suggest a structural correlation between the distribution of intraplate stresses, the thermal configuration of the plate and plate-boundary forces.

Geological inheritance has long been recognised to exert a first-order control on orogenesis^19–21^. The tectonothermal age of a subducting continental lithosphere has been proposed to control the strength of the orogenic lithosphere^19,21^. The spatio-temporal distribution of Heat Producing Elements (HPE) in the crust has also been shown to control the heat flux and local thermal structure in continental regions^22–25^. Chemically, the continental crust is enriched in HPE content with respect to the deeper mantle, especially in its upper and silica-rich parts (see Supplementary Fig. S1 online). Hence, a thickened orogenic crust is also associated with a higher than average HPE content^7,8^. Such a thermochemical configuration has a direct influence on the thermal and mechanical state of the lithosphere, therefore, on its mode of deformation during orogenesis. Within the AHCZ, despite observed differences in the timing of collision, crustal thickening beneath major orogens is a common characteristic. Indeed, the upper crust is thick in the Mesozoic-Cenozoic regions compared to older regions (see Supplementary Fig. S1 online). Similarly, the crust is thicker in all active regions, reaching as high as ~ 80 km beneath Tibet (see Supplementary Fig. S1 online). It is known that the end-member evolution of an orogen is to either lose its topography through erosion^26^ or to generate a new oceanic basin via gravitational collapse^27^. Previous studies using idealised physical models have addressed the interplay of erosion-sedimentation, tectonic thickening-thinning, thermal relaxation, and gravity-driving flow on processes such as runaway extension and necking instabilities in continental deformation^21,28^. In this study, we make use of information gathered on crustal thickness, age and chemical composition as a metric to estimate the stability and evolution of the continental lithosphere in the AHCZ. We investigate whether a causal correlation may be drawn between the thermomechanical state of the continental lithosphere, controlled by its crustal composition and thickness, and active plate-boundary forces. We further explore the geodynamic implications of such correlation with respect to the evolution of the continental lithosphere on Earth.

## Thermomechanical state of the lithosphere

We estimate the thermomechanical state of the AHCZ lithosphere (down to a reference depth of 200 km) by computing the minimum energy required to deform the lithosphere over geological time scales. Therefore, we first compute 3D steady-state temperature distribution in the AHCZ considering variations in the crustal layers as obtained from the Crust1.0^29^ model with representative radiogenic heat production and thermal properties (Table 1 and “Methods” Sect. 1). We use a constant temperature boundary condition of 15°C at the surface, while the temperature at the base has been derived based on a seismic tomography model (see “Methods” Sect. 2 and see Supplementary Fig. S2 online). We then compute the differential stress distribution for the AHCZ using equilibrium 3D temperature distribution and laboratory derived rheological properties representative for each layer in the model (see “Methods” Sect. 3 and Table 1). Thinner/thinned crustal regions (e.g., oceanic crust and back-arc basins in the Mediterranean) are overly weaker than stable continental interiors (Fig. 1a). The thickened crust beneath all major AHCZ orogens is also weaker than the crust beneath continental interiors, an observation consistent with the findings from previous studies^30^. On the scale of the whole lithosphere, domains corresponding to a thinner/thinned crust share a stronger lithosphere than continental interiors (Fig. 1b). These results are consistent with the fact that in Phanerozoic continental intraplate regions, the crust bears the bulk of stress, thereby allowing for efficient lateral transmission of plate-boundary forces to the adjacent orogens^31,32^.

**Table 1:** Thermophysical and rheological properties used for the different layers in the model.

Lithology	Density (kg/m^3^)	Thermal conductivity (W/Km)	Radiogenic heat production (W/m^3^)	Power-law strain rate A (Pa^−n^ s^−1^)	Power-law exponent n	Activation enthalpy H (J mol^−1^)
Sediments (Wet Granite^55^)	2400	2.5	1.0E−06	7.94 E−16	1.9	1.37E+05
Upper-crust (Dry quartz diorite^55^)	2750	2.4	1.65E−0.6	5.02 E−18	2.4	2.19E+05
Middle-crust (Dry quartz diorite^55^)	2850	2.1	0.5E−0.6	5.02 E−18	2.4	2.19E+05
Lower-crust (Dry mafic granulite^57^)	2950	2.0	0.2E−0.6	8.83 E−22	4.2	4.45E+05
Mantle (Dry peridotite^56^)	3300	3.0	0.01E−0.6	5.01 E−17	3.5	5.35E+05

The lithosphere beneath all orogens, despite the presence of cold subducted foreland lithosphere, such as beneath Tibet and Zagros, is overly weaker because of a thickened crust and higher than average HPE content (Fig. 1b, Supplementary Figs. S1, S2 online). The role of crustal heat production in buffering the mechanical state of the plate is also evidenced by the presence of a stronger lithosphere in the eastern European shield and western and eastern Africa, where crustal thickness is close to the global average (Fig. 1b, see Supplementary Fig. S1 online). We note an east–west increase in the strength of the orogenic lithosphere (from Tibet and Zagros to the Alps and Betics), which spatially correlates with a westward decrease in convergence velocities with respect to Eurasia^33^, and with an eastward crustal thickening and associated increase in crustal HPE content (see Supplementary Fig. S1 online).

## Thermochemical state of the crust and active deformation

The strength of the continental crust, modulated by the availability of its HPE content, has been discussed as the major controlling factor driving crustal thickness growth during the Archean^34^. Here, we discuss whether such a genetic relationship between strength, crustal composition and thickness can be extended to the Phanerozoic domains of the AHCZ. For a lithosphere of Mesozoic-Cenozoic age, that is encompassing tectonically active regions in the AHCZ, we observe a positive correlation between crustal strength and thickness up to a critical crustal thickness (C_r_ =  ~ 30–42 km). This critical value of crustal thickness is consistent with elevations of a few 100's meters as representative of the mean elevation of continental crust (Fig. 1c). From this point onward, the trend reverses and any further increase in crustal thickness is associated with crustal weakening. From our analysis, we can relate the observed decrease in crustal strength above C_r_ as being due to an increase in the concentration of HPE, which acts as the main dissipative source to limit crustal thickness to grow unbounded. This observation is robust despite the tectonothermal age of the crust considered (Fig. 1d). The actual value of C_r_ depends primarily on the assumed composition of the crust (see Supplementary Figs. S3, S4 online). Accounting for experimental uncertainties in rheological flow laws and their parameters, as well as our incomplete knowledge of the deeper crust and uncertainties of the Crust1.0 model, we found that the range of values of C_r_ resembles the global average continental crustal thickness^35^ (see Supplementary Figs. S3, S4 online).

**Figure 2:**
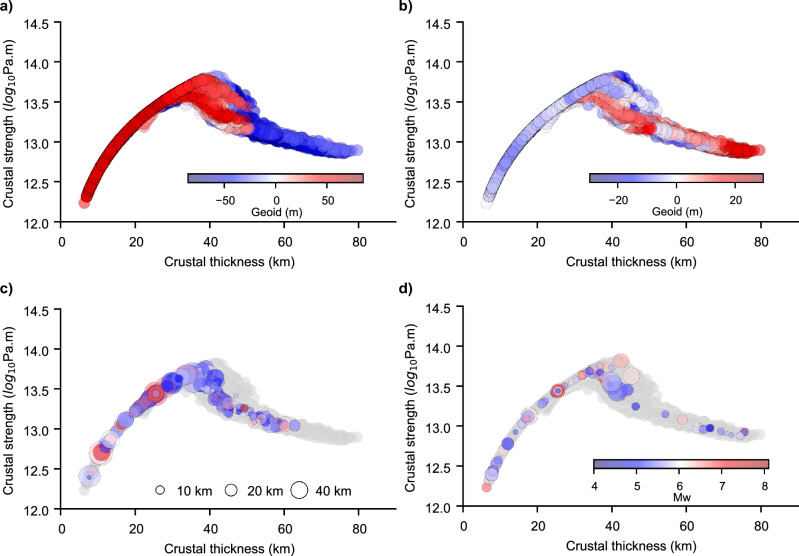
Geoid anomaly, seismicity, and crustal configuration. (**a**) Crustal strength versus crustal thickness colour coded by the geoid height from ICGEM using the GECO model^79^. In (**b**), the geoid height is filtered to a degree and order 10 (removing wavelengths higher than ~ 4000 km) to retain the signal from the upper mantle only. Note that the colour scale is centred at 0 m with red colours indicating positive values, and blue colours negative values. In the lower panels (**c**) and (**d**), earthquake epicentres at a given crustal thickness, are scaled by their hypocentre depths (≤ 80 km) (see legend in **c**) and colour-coded by their moment magnitudes (see legend in **d**)^80^. In (**c**) earthquakes with pure-thrust fault-plane solutions whereas in (**d**) earthquakes with pure-normal are plotted.

Regions with a crustal thickness higher than C_r_, are also characterised by a higher topographic relief, and therefore they store a higher gravitational potential energy than their surrounding domains (Fig. 1c,d). Filtering the geoid signal to isolate the contribution from the deeper mantle, we further notice that positive geoid anomalies overlap spatially with regions characterized by a thicker and weaker crust (Fig. 2a,b, Supplementary Fig. S5 online). This entails that the gravitational potential in the orogenic signal is acquired from the active plate-boundary forces contributing to the source of intraplate stress. It follows that a thicker than C_r_, and hence weaker orogenic crust, is likely to show a dominant extensional regime^21^ (Fig. 2c,d, Supplementary Fig. S5 online). This is consistent with the prevalence of normal fault plane solutions compared to thrust fault stress regime, which would instead favour crustal thinning via crustal flow^36–38^.

## Stability and evolution of the orogenic lithosphere

Plate tectonic models of continental growth agree on a critical behaviour of the continental lithosphere, which is characterized by alternating episodes of extension and compression in response to perturbations in the internal energy of the plate^39–41^. The picture that emerges from the AHCZ is that of a thermodynamically structured continental lithosphere evolving around a stable state. This stable state is controlled by the thermochemical configuration of the crust and is modulated by temperature-sensitive dissipative feedback mechanisms in response to plate-boundary forces; any variation in plate-boundary forces would cause a change in the thermodynamic state of the evolving plate and move the system out of equilibrium, i.e. to a state of non-zero internal energy. This simple thermodynamic model enables us to interpret observed crustal thickness variations in the AHCZ as finite-amplitude perturbations from a stable energetic state in equilibrium with active plate-boundary forces. To describe the geodynamics of the continental interiors, it is, therefore, crucial to properly trace the dissipation path and quantify the magnitudes and rates involved in the process. In doing so, we follow previous studies^42^ and graphically represent the dissipative evolution via a phase space described in terms of the mean lithosphere temperature and crustal thickness configuration (see “Methods” Sect. 4). We find that within this phase space, C_r_ represents a fixed-point attractor and any external force, i.e. finite-amplitude perturbation, engenders variations in rates of crustal growth (thickening or thinning). This in turn triggers the onset of a dissipative thermo-mechanical feedback loop steering the evolution of the system (see “Methods” Eqs. 4.1, 4.2, 4.7).

**Figure 3:**
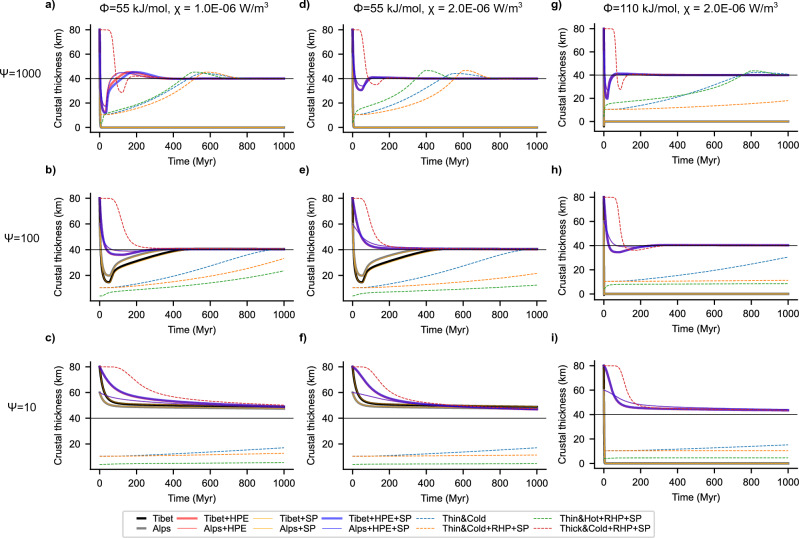
Stability and evolution dependence on energy parameter. Each panel shows the evolution of crustal thickness for possible initial states, also representing scenarios found in the major orogens of AHCZ. The black and grey curves denote experiments with initial state that resembles the lithosphere beneath Tibet and Alps, with no heat production in the crust, respectively. The orange curves illustrate the additional effect of considering surface processes (erosion and sedimentation) with a constant rate of 10 mm/yr. Note that there is only a slight difference by including surface processes, hence, orange curves overlay. Red curves consider the effect of heat production ($\upchi$ indicated in the panel title) for the perturbations equivalent to Tibet (thicker curves) and Alps (thinner curves). Similarly, the blue curves consider the additional effect of surface processes with a constant erosion and sedimentation rate of 10 mm/yr. Coloured dashed curves represent experiments where the initial state has been changed to sample other end-member perturbations. Each row represents an experiment with different $\uppsi$ (see the row title), and each column with different creep activation energy, $\upphi$, and HPE, $\upchi$ (see the column title). Decreasing the ratio of the thermal to viscous relaxation time, the run-away extensional states with no HPE, are attracted towards the equilibrium but take longer time (top to bottom). Decreasing the energy parameter leads to overall higher viscosities and lower strain-rate (Fig. 4), preventing these states from going to the run-away extension. Increasing the HPE and activation energy (left to right) increases the overall viscosity leading to dampening of the oscillations and shorter time to attain equilibrium crustal thickness.

**Figure 4:**
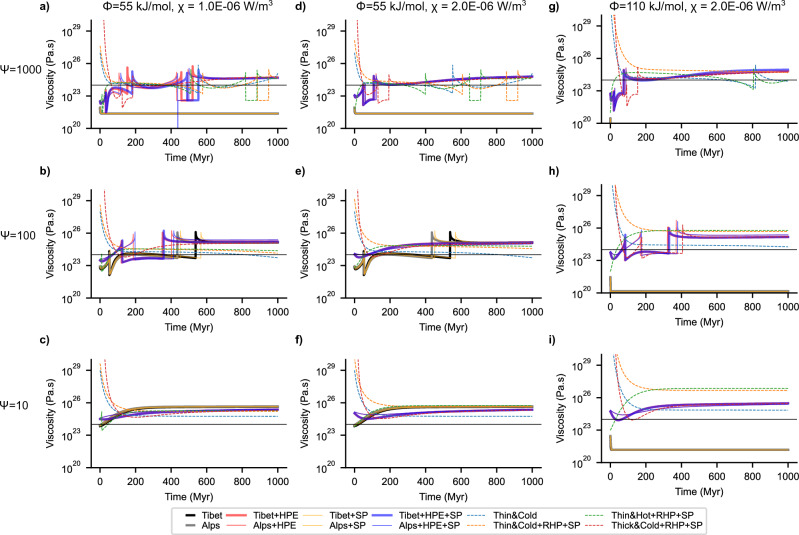
Stability and evolution continental lithosphere. Viscosity evolution for different initial states corresponding to the crustal evolution in Fig. 3. Decreasing the ratio of the thermal to viscous relaxation time, the run-away extension states with no HPE, are attracted towards the equilibrium (top to bottom). Decreasing the energy parameter leads to overall higher viscosities and lower strain-rate, preventing these states from going to the run-away extension. Increasing the HPE and activation energy (left to right) increases the overall viscosity. Note that the experiments with $\uppsi$=1000 (top row) show viscosities appropriate to the continental lithosphere.

**Figure 5:**
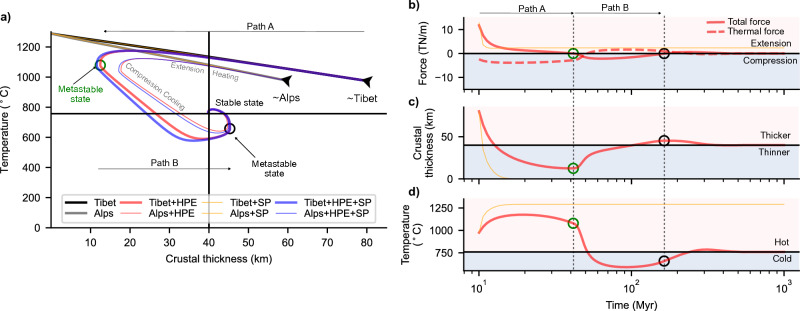
Stability and evolution of the orogenic lithosphere. (**a**) Phase diagrams in crustal thickness-temperature space for orogen style perturbations (indicated by arrows) representing crustal thickness and temperatures found in the major orogens of AHCZ. The physical parameters are the same as in Figs. 3a and 4a. The black and grey curves denote experiments with initial state that resembles the lithosphere beneath Tibet and Alps, with no heat production in the crust, respectively. The orange curves illustrate the additional effect of considering surface processes (erosion and sedimentation) with a constant rate of 10 mm/yr. Note that there is only a slight difference by including surface processes, hence, orange curves overlay the black and grey curves. Red curves consider the effect of heat production (H of 1.0E−06 W/m^3^) for Tibet (thicker curves) and Alps (thinner curves) like states. Similarly, the blue curves consider the additional effect of surface processes with a constant erosion and sedimentation rate of 10 mm/yr. In (**b**)–(**d**) we only show time evolution of driving force, crustal thickness and mean lithospheric temperature corresponding to Tibet-type perturbation with (red curves) and without HPE (orange curves). In (**b**) total driving force evolution with time (solid red: with HPE) and contribution to it from thermal evolution (dashed red) of the system. Orange curve in (**b**) shows the evolution of total energy for a state with no HPE that shows runaway extension. In (**c**) and (**d**) we plot the corresponding evolution in time of the crustal thickness and mean lithospheric temperature, respectively. Open green and black circles in all the plots denote points where total force is zero within the first 200 Myr of the evolution referred to as metastable states. Paths marked as A and B represent key stages in the evolution of these experiments (see text for details).

The exact characteristics of the evolutionary path depend on both the amplitude of the initial perturbation, the source of the initial driving energy, and the relaxation time scale of the active dissipative process, whether thermal diffusion and/or viscous deformation. We can quantify the resulting evolution of the plate via a single non-dimensional constant ($\uppsi$), the ratio of the timescale of thermal diffusion to viscous relaxation, which provides an estimate of the internal energy of the evolving system. Typical ranges of thermal properties and viscosities of the continental lithosphere provide a lower bound to such parameter, that is $\uppsi \ge 1$. In addition, oscillatory cycles around a fixed-point attractor can only arise if the system passes through metastable states, that is states of null total energy but with non-zero thermal and mechanical contributions (Figs. 3, 4, 5, “Methods” Eqs. 4.5–4.7). These states can only occur if the thermal and viscous relaxation time scales differ, thereby imposing a second condition on $\uppsi$ that is, $\uppsi >$ 1. In addition, $\uppsi =$ 1 implies overly stiff plates which is not representative of Earth featuring mobile-lid plate-tectonics.

If we consider magnitudes of perturbations as found in the AHCZ (see Supplementary Fig. S6 online), for a linear Newtonian viscosity of the lithosphere, we observe decaying oscillatory behaviour only for a limited range of the energy constant $(1<\uppsi$ < 1000, see Supplementary Fig. S7 online). Considering a more realistic, non-Newtonian viscosity configuration, the width of the decaying oscillatory domain is additionally limited by the activation energy of the material considered (Figs. 3, 4, Supplementary Fig. S8 online). Surface processes (erosion and sedimentation) do not change the general behaviour of the system. The isostatic response of the lithosphere to this ancillary source of deformation acts instead to restore the long-term equilibrium state of the continental plate (Fig. 5a red and blue curves). By decreasing the energy constant $\uppsi$ (e.g. by considering higher effective plate viscosities) we observe further dampening of the oscillations leading to an exponential-like decay in crustal thickness over longer time (Figs. 3, 4, Supplementary Figs. S9, S10 online). As discussed above, the limiting case of $\uppsi$=1 rules out any metastable state and portrays a path decaying exponentially to the final attractor point. Runaway extension, in the form of exponentially growing oscillations, are only possible at high energy states (e.g., $\uppsi \ge$ 1000, Figs. 3, 4). However, they can be effectively damped out by thermally driven dissipative processes. The main source of dissipation lies in the additional heat source from HPE in the crust (Fig. 5a). Neglecting this contribution results in relatively high initial strain-rates, low effective viscosity and in crustal thinning at rates faster than the diffusive time scale of the plate (Fig. 5c). Under these conditions, the system is able to maintain a finite amount of internal energy throughout its evolution, which supports the ongoing crustal thinning finally leading to run away and rift-like extension (Fig. 5a, thin yellow, and black and grey lines). The contribution from HPE is to increase the amount of energy being dissipated in the system (see “Methods” Eq. 4.6: second term in right) thereby lowering the driving force to crustal thinning (Fig. 5b; “Methods” Eqs. 4.5–4.7). As a result, the system undergoes crustal thinning at lower rates (Path A in Fig. 5c). Enhanced dissipation rates from HPE also affect the energetic balance, with the plate gradually losing the driving internal energy upon reaching a first metastable state of null energy (Fig. 5 green open circle; also see “Methods” Eqs. 4.5, 4.6). This metastable state is characterized by a balance between extensional forces from viscous creep and increasing compressional forces from thermal cooling. This metastable state is also characterized by a crust that has yet to attain its critical thickness, being thinner and hotter (Fig. 5c,d). Ongoing thermal diffusion leads to plate cooling and crustal thickening associated to the onset of a compressional regime. Cooling and associated crustal thickening continue until reaching a point where heat from the increasing HPE concentration starts to partially compensate for the ongoing cooling (Path B in Fig. 5), leading to lower rates of crustal thickening and a reverse thermal trend. From this point onward, crustal thickening is accompanied by heating at gradually higher rates, and the system passes through a second metastable state of zero internal energy (black circle in Fig. 5). This state is characterized by a crust thicker and colder than C_r_, therefore, the system undergoes another cycle of extension-heating followed by cooling-thickening, similar to the one described above. Such cycles of extension and compression are driven by the available energy in the system, i.e., non-zero thermal and/or mechanical energy. As such, their amplitudes are a function of the magnitude of the initial perturbation (Fig. 5a, Alps vs. Tibet, and Fig. 3, dashed curves) as well as the non-dimensional energy constant (see Supplementary Fig. S9 online). The frequency of each oscillation (distance in time between metastable states) depends on the ability to dissipate the available energy that fuels its dynamics. By increasing the HPE concentration (promoting higher dissipation) metastable states lie closer to each other in time, thus lowering the frequency of the oscillations. The limiting case of relatively high HPE content is associated with an „overdamped” system evolving in time to the final equilibrium crustal thickness without passing through metastable states (i.e., no oscillations) (Fig. 5, Supplementary Fig. S10 online), while for the other end-member case of no HPE, the system showcases exponentially growing oscillations and runaway rift-like extension.

Our results show that the high amplitude perturbations to the equilibrium state, as typified in orogens and plateaus in the AHCZ, could be dissipated to attain crustal thickness similar to continental interiors, suggesting a potential mechanism for craton formation, or, depending on the effective role of surface processes, formation of an intracratonic basin^13^ (see also Figs. 3, 4, 5). A thicker than average continental crust will be subjected to an extensional stress state leading to crustal thinning in response to strain accumulation, and to a time-average dissipation of its potential energy. We additionally found that the time taken to attain such an equilibrium is in the order of a few 100’s Myrs, a time scale that is consistent with estimated seismogenic strain rates in the AHCZ and other continental interiors^17,33^. The regime of exponentially growing oscillations is associated with a state of extensional forces of the order of plate driving forces (~ 10^12^N/m) (Fig. 5b, Supplementary Fig. S10 online). Such a state is thermodynamically unstable, and therefore it will evolve over time to reach thermodynamic equilibrium via passively thinning the underlying mantle, resembling the dynamics driving passive rifting^43^.

The lithosphere-asthenosphere system has been shown to be characterised by a marginal stability^44^. This being true, it follows that any perturbation to its initial state from the deeper convective mantle could exert an additional influence on its dissipative evolutionary path. If we consider end-member values for mantle convection velocities and length scales^45,46^, we compute mantle transit time scales in the order of ~ 100 Myrs (see Supplementary Fig. S11 online). These time scales are in the same order of magnitude as computed relaxation time scales of the lithosphere (Figs. 3, 4, 5). We, therefore, suggest that deep mantle perturbations, in the form of plume dynamics or mantle flow driven necking instabilities^28,47^, can additionally modulate the system behaviour, potentially leading to runaway extension and the formation of a new oceanic basin.

## Stable equilibrium dynamics: plate-tectonics and beyond

**Figure 6:**
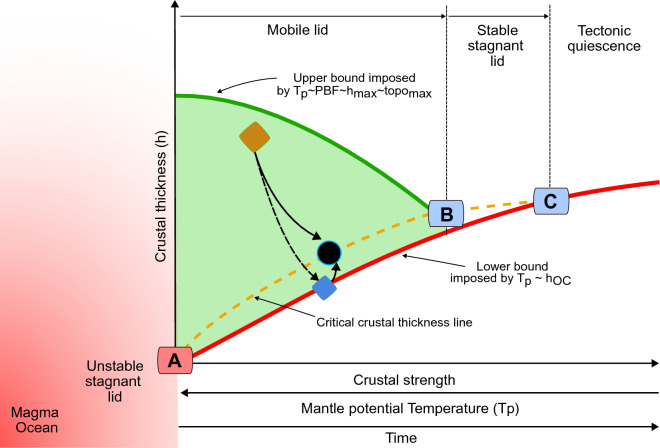
Conceptual model for plate-tectonics. Schematic diagram depicting the evolution of an Earth-like planet in terms of its crustal thickness (h), crustal strength, and mantle potential temperature (T_p_). The onset of plate tectonics is marked by point A after initial accretion (red domain to the left). The red curve denotes the secular cooling in terms of T_p_ (decreasing to the right), which controls the composition and thickness of the oceanic lithosphere (h_oc_), thus setting a lower bound to the stability domain of mobile-lid plate tectonics. The dark green curve is the upper bound on crustal thickness growth during orogenesis, resulting from stresses imparted to the lithosphere from plate-boundary forces (PBF). The light green area in between represents the possible parameter space in terms of crustal thickness that may evolve in response to PBF and prevailing T_p_. The dashed orange curve denotes the critical crustal thickness in equilibrium with PBF, as dictated by T_p_ and the internal energy of the crust. Point B represents the onset of a stable stagnant lid tectonic regime, in which subduction ceases, representing mechanical equilibrium between driving and resisting forces favouring thermal relaxation via diffusion. Point C denotes a stage of thermal equilibrium between the crust and mantle, marking tectonic quiescence. Any perturbation to the stable equilibrium state (black circle), such as thickening during orogenesis (orange diamond) or thinning (blue diamond), cause the system to evolve around this stable equilibrium state, the path of which is a function of the crustal rheology, composition, and internal energy content. The solid black arrows illustrate a typical evolutionary path from orogens to ocean lithosphere, whereas the dashed arrow indicates a system that goes towards runaway extension (rifting).

Previous studies have noted a temporal correlation between the onset of plate tectonics on Earth and the degree of crustal differentiation, from a mafic dominated to a silica-enriched composition, and associated thickening of continental crust^48^ (point A in Fig. 6). Crustal strength is an expression of the active tectonic forces and the internal energy of the plate. By neglecting the additional contribution from HPE, it follows that any increase in crustal thickness above C_r_ (Fig. 1c,d) would therefore be associated to an increase in the plate strength. Such a situation would be consistent with a stable stagnant lid behaviour rather than mobile lid tectonics^49^. Therefore, for plate tectonic to exist, it requires a physical equilibrium between the thermochemical state of the continental plate and prevailing plate-boundary forces arising from mantle dynamics (orange curve and black circle in Fig. 6). We have shown that such an equilibrium is characterized by a critical crustal thickness reflecting its thermochemical state, which, given present-day plate-boundary forces, matches the global average of continental crust on Earth.

The mantle potential temperature (T_p_) is widely accepted as a measure of the energy empowering plate tectonics, by controlling the thickness and composition of oceanic lithosphere and resulting negative buoyancy of subducting slabs. The secular cooling of the ambient mantle is thought to influence the coupling across the lithosphere-asthenosphere boundary, the density contrast between the lithospheric and convective mantle, as well as lithospheric thickness. It follows that the evolution of T_p_ provides a limiting factor to the critical crustal thickness and therefore to the existence of plate tectonics on Earth. Higher than present-day T_p_ values would imply a warmer Earth, with a thinner and weaker C_r_, promoting an unstable stagnant lid behaviour, characterized by recurrent material recycling, large-scale lithospheric failure, and widespread magmatism, or, in the limiting case of zero C_r_, generation of a primordial magma ocean (Fig. 6). In contrast, lower than present-day T_p_ values, as expected from the ongoing secular cooling, would imply a higher C_r_ and therefore a stronger lithosphere. This configuration would additionally be associated with a decrease in plate-boundary forces, and with time, with a state of mechanical equilibrium between lithospheric strength and stresses associated with plate-boundary forces (point B in Fig. 6). This would give rise to a system that will cool preferentially by diffusion, thus leading to the formation of thick and stable stagnant lids, and, in the limiting case of unbounded crustal growth, to tectonic quiescence (point C in Fig. 6). Therefore, we conclude that the presence of a critical crustal thickness on a cooling Earth-like planet represents a necessary condition for plate tectonics to occur. Further, such a critical crustal thickness is crucial to maintain the average height of continents above sea level, thereby controlling the distribution of water on continents as relevant for the origin of life^50,51^ and bio-diversity^52^. Given the limitations imposed on crustal growth by the internal HPE contribution together with the ongoing secular mantle cooling, a stable stagnant lid is the most likely scenario for the future Earth, thus setting a “time limit” to plate tectonics as we observe today.

## Methods

### 3D structural model of the lithosphere

The structural model of the lithosphere in the Alpine Himalayan Collision Zone (AHCZ), consists of sediments, upper-crust, middle-crust, lower-crust and lithospheric mantle. Crustal layer thicknesses are taken from the Crust1.0 model^29^ with respect to the ETOPO1 digital elevation model^53^, and are interpolated and resampled at 0.5 degree grid resolution which is the horizontal resolution of the 3D structural model (see Supplementary Fig. S1 online). The base of the model is set at a depth of 200 km, encompassing the lithospheric mantle. The tectonothermal age of the crust is taken from the compilation by Goutorbe et al.^54^ allowing to differentiate the crustal layers according to their tectonothermal age (see Supplementary Fig. S1 online). Thermophysical and rheological properties in the layers to compute temperature (Sect. 2) and strength (Sect. 3) are assigned according to the representative lithologies^23,55–57^ of the layers and are listed in Table 1 and Table S1.

### Temperature distribution

Temperature in the 3D structural model is computed assuming steady-state. The conductive thermal field, including the radiogenic heat production, is computed using:

2.1 $$\nabla \cdot \left( {{\text{k}}\nabla {\text{T}}} \right) + {\text{H}} = 0$$

where $\nabla$ is the del operator, $\mathrm{T}$ is the temperature, $\mathrm{k}$ is the thermal conductivity, and $\mathrm{H}$ is the radiogenic heat production in the different layers of the model (Table 1). We take a fixed boundary of 15 °C at the surface and a fixed temperature boundary condition at the base (200 km) derived from the conversion of shear-wave tomography models to temperature (see below). Equation 2.1 is solved numerically in 3D using finite-elements software GOLEM^58^.

Shear-wave velocities (Vs) at 200 km depth^59^ are converted to temperature using Gibbs free-energy minimisation algorithm, for details see^16^. We pre-compute anharmonic Vs from stable phase and mineral assemblages at upper-mantle pressure and temperature conditions, computed using a Gibbs free-energy minimisation algorithm^60^, and depleted-mid-oceanic-ridge-basalt-mantle (DMM^61^) as bulk composition. Pressure and temperature-dependent anharmonic Vs are corrected for anelasticity using parameters derived from laboratory experiments^62^:

2.2 $${\text{V}}_{{\text{S}}} = {\text{V}}_{{{\text{So}}}} \left( {\text{T, P}} \right)\left[ {1 - \frac{1}{2}\cot \left( {\uppi \frac{\upalpha }{2}} \right){\text{Q}}_{{\text{S}}}^{ - 1} } \right]$$

where V_So_(T, P) is the anharmonic shear-wave velocity at a given temperature and pressure, and ${\text{Q}}_{\text{S}}^{-{1}}$ is the anelastic correction factor and is expressed as^62^:

2.3 $${\text{Q}}_{{\text{S}}}^{ - 1} \left( {\upomega ,{\text{T}},{\text{P}},{\text{C}}_{{{\text{OH}}}} ,{\text{d}}} \right) = \left[ {{\text{Bd}}^{{ - {\text{p}}_{{\text{Q}}} }}\upomega ^{ - 1} \exp \left( { - \frac{{\left( {{\text{E}}_{{\text{Q}}} + {\text{V}}_{{\text{Q}}} {\text{P}}} \right)}}{{{\text{RT}}}}} \right)} \right]^{\upalpha }$$

2.4 $${\text{B}} = {\text{B}}_{{\text{o}}} {\text{d}}_{{{\text{Qref}}}}^{{{\text{p}}_{{\text{Q}}} - {\text{p}}_{{{\text{Qref}}}} }} \left( {\frac{{{\text{C}}_{{{\text{OH}}}} }}{{{\text{C}}_{{{\text{OH}}\left( {{\text{Qref}}} \right)}} }}} \right)^{{{\text{r}}_{{\text{Q}}} }} \exp \left( {\frac{{\left( {{\text{E}}_{{\text{Q}}} + {\text{P}}_{{{\text{Qref}}}} {\text{V}}_{{\text{Q}}} } \right) - \left( {{\text{E}}_{{{\text{Qref}}}} + {\text{P}}_{{{\text{Qref}}}} {\text{V}}_{{{\text{Qref}}}} } \right) }}{{{\text{RT}}_{{{\text{Qref}}}} }}} \right)$$

where $\upomega$ is frequency, ${\text{C}}_{{{\text{OH}}}}$ is olivine water concentration expressed in H/10^6^Si, d is the grain size, B is the pre-exponential constant defined in Eq. 2.4, ${\text{E}}_{{\text{Q}}}$ = 420 kJ/mol is the activation energy, ${\text{V}}_{{\text{Q}}}$ = 1.2 × 10^−5^ m^3^/mol is the activation volume, R = 8.314 JK^−1^ mol^−1^ is the universal gas constant, p_Q_ is the grain size exponent, and $\upalpha$ is the frequency dependence factor. The reference values, ${\text{p}}_{{{\text{Qref}}}}$, ${\text{C}}_{{\text{OH(Qref)}}}$, ${\text{E}}_{{{\text{Qref}}}}$, ${\text{V}}_{{{\text{Qref}}}}$, ${\text{P}}_{{{\text{Qref}}}}$, ${\text{T}}_{{{\text{Qref}}}}$ are taken from Behn et al.^62^. Effects of partial melts are incorporated by computing an indicative measure of melt fractions using empirical dry-peridotite solidus (${\text{T}}_{{\text{m}}}^{{\text{s}}}$) and liquidus (${\text{T}}_{{\text{m}}}^{{\text{l}}}$), both in °C, as a function of pressure (${\text{P}}$) in GPa after^63–65^ as:

2.5 $${\text{T}}_{{\text{m}}}^{{\text{s}}} = 1080 + 134.2{\text{P}} - 6.581{\text{P}}^{2} + 0.1054{\text{P}}^{3}$$

2.6 $${\text{T}}_{{\text{m}}}^{{\text{l}}} = 1762 + 57.46{\text{P}} - 3.487{\text{P}}^{2} + 0.077{\text{P}}^{3}$$

The indicative melt fraction, ${\text{M}}$, at a given temperature and pressure is calculated as:

2.7 $${\text{M}} = \frac{{\left( {{\text{T}} - {\text{T}}_{{\text{m}}}^{{\text{s}}} } \right)}}{{\left( {{\text{T}}_{{\text{m}}}^{{\text{l}}} - {\text{T}}_{{\text{m}}}^{{\text{s}}} } \right)}}$$

where ${\text{T}}$ is the temperature at which anharmonic shear-wave velocities are calculated from the Gibbs-free energy minimisation algorithm. Anharmonic shear-wave velocities are corrected for the effects of partial melts using the empirical relation ${\text{dInVs/(\% melt)}} = - {5}{\text{.3}}$, such that per percentage of melt fraction decreases shear-wave velocities by 5.3%^66,67^. The absolute Vs values from the tomography models are then projected onto the pre-computed look-up table of Vs using a lithostatic pressure profile derived from a thermochemical equivalent model of ak135^68,69^. We also tested the effect of lower temperature boundary condition derived from different seismic tomography models on the strength (see Supplementary Fig. S4 online).

### Strength

The long-term yield strength in the 3D model is calculated by computing the maximum differential stress before permanent deformation^70,71^. Frictional behaviour is assumed at shallow depths described by Byerlee’s law^72^:

3.1 $$\Delta\upsigma _{{\text{b}}} = {\text{f}}_{{\text{f}}}\uprho _{{\text{b }}} {\text{gz}} \left( {1 - {\text{f}}_{{\text{p}}} } \right)$$

where $\Delta\upsigma _{{\text{b}}}$ is the brittle yield strength, ${\text{f}}_{{\text{f}}}$ is the Byerlee’s friction coefficient which depends on the internal friction coefficient ${\text{u}}_{{\text{f}}}$ and the faulting regime (e.g., 3: compressional, 0.75: extensional, 1: strike-slip), $\uprho _{{\text{b}}}$ is the bulk density (see Table 1 and Supplementary Table S1 online), g is the gravitational acceleration, and z is the depth. The pore fluid factor, ${\text{f}}_{{\text{p}}}$, is considered constant with a value of 0.36, representing near hydrostatic conditions.

With increasing depth, temperature increases and rocks tend to deform through thermally activated processes manifesting as a viscous (non-) Newtonian fluid, with dislocation creep being the dominant deformation mechanism in the lithosphere. Here, we also included low-temperature plasticity (Peierl’s creep) at differential stresses greater than 200 MPa^73,74^. The power laws describing dislocation creep (Eq. 3.2) and low-temperature plasticity (Eq. 3.3) are expressed as:

3.2 $$\Delta\upsigma _{{\text{d}}} = \left( {\frac{{{\dot{\upepsilon}} }}{{{\text{A}}_{{\text{p}}} }}} \right)^{{\frac{1}{{\text{n}}}}} \exp \left( {\frac{{\text{H}}}{{{\text{nRT}}}}} \right)$$

3.3 $$\Delta\upsigma _{{\text{D}}} =\upsigma _{{\text{D}}} \left( {1 - \left[ { - \frac{{{\text{RT}}}}{{{\text{Q}}_{{\text{D}}} }} \ln \frac{{{\dot{\upepsilon}} }}{{{\text{A}}_{{\text{D}}} }}} \right]^{\frac{1}{2}} } \right)$$

where $\Delta\upsigma _{{\text{d}}}$ is the ductile yield strength, ${\dot{\upepsilon}}$ is the reference strain rate taken to be 1E−15 s^−1^^33^, ${\text{A}}_{{\text{p}}}$ is the pre-exponential scaling factor for dislocation creep, ${\text{n}}$ is the power law exponent, ${\text{H}}$ is the creep activation enthalpy, T is the temperature, and R is the universal gas constant. Values of these parameters for different lithological layers are listed in Table 1. In Eq. 3.3, $\Delta\upsigma _{{\text{D}}}$ is the Peierls’s critical stress, ${\text{Q}}_{{\text{D}}}$ is the Dorn activation energy (5.35E5 Jmol^−1^) and ${\text{A}}_{{\text{D}}}$ is the Dorn’s law strain rate (${\text{A}}_{{\text{D}}}$ = 5.7E11 s^−1^). The yield strength ($\Delta\upsigma _{\max }$) at a given point is defined by the minimum of the brittle and ductile strength, $\Delta\upsigma _{{\text{b}}}$ and $\Delta\upsigma _{{\text{d}}}$^71^. The integrated strength, crustal and lithospheric, is calculated by integrating strengths over corresponding depths. We also tested other reported rheological laws for the representative lithologies, Table S1 and Fig. S3.

### Stability and evolution of the continental lithosphere

Evolution of the continental lithosphere away from the equilibrium is driven by the energy difference with respect to the equilibrium column, and its dissipation through the thermal and mechanical relaxation. It is described as a function of two state variables: the thickening/thinning factor f = C/C_r_, which describes the thickness of the crustal layer, C, with respect to an equilibrium column with crustal thickness C_r_ taken to be 40 km (i.e., stable state), and the mean temperature of the lithospheric column ${\overline{\text{T}}}^{{\prime }}$, for details see^42^. These state variables are coupled (Eqs. 4.1, 4.2) such that advection of heat is controlled by evolution of f (Eq. 4.1 i.e., extension or compression); hence it drives ${\overline{\text{T}}}^{{\prime }}$, and temperature-dependent gravitational potential energy (Eqs. 4.5, 4.6) controls extension or compression hence driving ${\text{f}}$ (Eq. 4.2). A feedback loop exists in which the evolution of the thermal state lags the induced strain because of a longer time required for thermal diffusion. Radiogenic heat production which is a function of crustal thickness also modulates the thermo-mechanical dissipative feedback loop via decreasing the temperature-dependent driving energy (Eq. 4.6).

The evolution of both the temperature and strain with respect to the equilibrium column is formulated as a function of f and ${\overline{\text{T}}}^{{\prime }}$ as:

4.1 $$\frac{1}{{\text{f}}}\frac{{{\text{df}}}}{{{\text{dt}}^{{\prime }} }} = - {\dot{\upepsilon}} ^{{\prime }}_{{{\text{xx}}}}$$

4.2 $$\frac{{{{{\text{d}}\overline{\text{T}}}}^{{\prime }} }}{{{\text{dt}}^{{\prime }} }} = - \left( {{\overline{\text{T}}}^{{\prime }} - \overline{{{\text{T}}_{c}^{{\prime }} }} } \right) + {\dot{\upepsilon}} _{{{\text{xx}}}}^{{\prime }} \left( {1 - {\overline{\text{T}}}^{{\prime }} } \right)$$

where ${\text{t}}^{{\prime }}$ is dimensionless time, ${\dot{\upepsilon}} _{{{\text{xx}}}}^{{\prime }}$ is the dimensionless horizontal strain rate, ${\overline{\text{T}}}^{{\prime }}$ is the dimensionless mean temperature, and $\overline{{{\text{T}}_{{\text{c}}} }}^{{\prime }}$ is the dimensionless mean temperature for the conductive part of the heat transport. In Eq. 4.2, the local transient geotherm is computed relative to the conduction geotherm using the principal Fourier mode where $\overline{{{\text{T}}_{{\text{c}}} }}^{{\prime }}$ accounts for the conductive part (Eq. 4.4) and the second term on the right (Eq. 4.2) accounts for the advective part of heat transport through the strain-rate. Constant vertical velocity with depth is assumed, and the column is considered to be in mechanical equilibrium in the horizontal direction, i.e., vertical and horizontal velocities are similar (i.e., pure shear). Non-dimensionalisation is performed for length using ${\text{L}}_{{\text{o}}}$ = 140 km which is the thickness of the equilibrium lithosphere, for time using ${\text{L}}_{{\text{o}}}^{{2}} {{\uppi }}^{{2}} {\text{K}}$ where **K** = 1.0E−06 m^2^/s is the thermal diffusivity, for temperature using T_L_ = 1300 °C which is the temperature at the base of the lithosphere, for density using a density of the mantle as ρ_m_ = 3300 kg/m^3^, and for stress, using ${\text{g}}{{\uprho }}_{{\text{m}}} {\text{L}}_{{\text{o}}}$, where ${\text{g}}$ is the gravitational acceleration. Dimensionless parameters that control the time evolution of the lithospheric column, with respect to the equilibrium column, are coupled to lithospheric thickness, temperature, potential energy, and strain rate leading to the following systems of equations:

4.3 $${\text{L}}^{{\prime }} = \left[ {1 + {\rm B}\Gamma \left( {{\text{f}} - 1} \right)} \right]\left[ {1 + \Omega \left( {{\overline{\text{T}}}^{{\prime }} - \overline{{{\text{T}}_{{{\text{cr}}}} }}^{{\prime }} } \right)} \right] + \Delta {\text{L}}^{{\prime }}_{{{\text{SP}}}}$$

4.4 $$\overline{{{\text{T}}_{{\text{c}}} }}^{{\prime }} = \frac{1}{2} + {\text{ f}}^{2} \frac{\upchi }{4}\left( {1 - \frac{2}{3}{\text{f}}\Gamma } \right)$$

4.5 $${\text{E}}^{\prime } = \frac{{\text{B}}}{2} \left[ {\left( {{\text{L}}^{{\prime }} - {\text{f}}\Gamma } \right)^{2} - \left( {1 - \Gamma } \right)^{2} } \right] + \frac{{\left( {1 - {\text{B}}} \right)}}{2}\left( {\text{L}^{{{\prime }2}} - 1} \right) + \Delta {\text{E}}^{\prime }$$

4.6 $$\Delta {\text{E}}^{{\prime }} = \frac{\Omega }{2} \left[ {\overline{{{\text{T}}_{{\text{c}}}^{{\prime }} }} + \overline{{{\text{T}}_{{{\text{cr}}}}^{{\prime }} }} \left( {{\text{L}}^{{{\prime }2}} - 1} \right) - {\overline{\text{T}}}^{{\prime }} \left( {2{\text{L}}^{{\prime }} - 1} \right) - \left( {{\text{L}}^{{\prime }} - 1} \right)^{2} } \right. - \left. {\frac{\upchi }{12}\left( {{\text{f}}^{2} \left( {2 - {\text{f}}\Gamma } \right)^{2} - \left( {2 - \Gamma } \right)^{2} } \right)} \right]$$

4.7 $${\dot{\upepsilon}} _{{{\text{xx}}}}^{{\prime }} = \uppsi \left( {\frac{{{\text{E}}^{{\prime }} }}{{{\text{L}}^{{\prime }} }}} \right)^{{\text{n}}} {\text{exp}}\left[ {\upphi \left( {\frac{{1}}{{\overline{{{\text{T}}_{{{\text{cr}}}}^{{\prime }} }} }} - \frac{{1}}{{{\overline{\text{T}}}^{{\prime }} }}} \right)} \right]$$

where Γ = C_r_/L_o_ is the crust to lithosphere thickness ratio of the equilibrium state, B = (ρ_m−_ρ_c_)/ρ_m_ is the crustal density ratio with a crustal density of ρ_c_ = 2800 kg/m^3^, Ω = ρ_m_αT_L_/ρ_m_ is the thermal buoyancy ratio with reference density ρ_o_ = 3260 kg/m^3^ and thermal expansion coefficient of α = 3.0E−05 °C^−1^, $\upchi = \frac{{{\text{H}}_{{\text{o}}} {\text{C}}_{{\text{r}}}^{2} }}{{{\text{ KT}}_{{\text{L}}} }}$ is the dimensionless heat generation rate where H_o_ = H/(ρ_c_C_p_), with the radiogenic heat production H, and specific heat C_p_ = 1000 Jkg^−1^ K^−1^, $\upphi = \frac{{\text{Q}}}{{{\text{RT}}_{{\text{L}}} }}$ is dimensionless activation energy with the activation energy for creep Q = 55 kJ/mol, R being the universal gas constant, ${\text{n}}$ is the rheological exponent, $\uppsi = \frac{{{\text{C}}_{{\text{o}}} {\text{L}}_{{\text{o}}}^{2} }}{{\uppi ^{2} {\text{K}}}} \left( {{\text{g}}\uprho _{{\text{m}}} {\text{L}}_{{\text{o}}} } \right)^{{\text{n}}}$ is the ratio of thermal to viscous relaxation timescales, where ${\text{C}}_{{\text{o}}} = \frac{{{\text{Q}}\uppi ^{2} {\text{K}}}}{{{\text{L}}_{{\text{o}}}^{2} }} \left( {{\text{g}}\uprho _{{\text{m}}} {\text{L}}_{{\text{o}}} } \right)^{{\text{n}}}$ is the strain-rate coefficient and $\overline{{{\text{T}}_{{{\text{cr}}}} }}^{{\prime }}$ = 650 °C is the mean temperature of the equilibrium state. The driving energy ${\text{E}}^{{\prime }}$ in Eq. 4.5 includes the mechanical contribution resulting from the thickness variation with respect to the equilibrium column (first term on the right), and thermal contribution ($\Delta {\text{E}}^{{\prime }}$). The thermal part of the energy includes the temperature difference with respect to the equilibrium column (Eq. 4.6: first term in the right) and heat contribution from the radiogenic heat production (Eq. 4.6: second term in the right).

Effect of surface processes, erosion, and sedimentation, is incorporated as a correction to the available potential energy (${\text{E}}^{{\prime }}$) through the column thickness, via an additional term $\Delta {\text{L}}^{{\prime }}_{{{\text{SP}}}}$ (Eq. 4.3). If the crustal thickness is higher than the equilibrium crustal thickness, erosion is set with a specified constant rate, whereas below this, sedimentation is set with a specified constant rate. Hence, erosion/sedimentation is scaled by the height difference with respect to the equilibrium crustal thickness as:

4.8 $$\Delta {\text{L}}^{{\prime }}_{{{\text{SP}}}} = \left\{ {\begin{array}{*{20}c} { - {\text{k}}^{{\prime }}_{{{\text{erosion}}}} \left( {{\text{L}}^{{\prime }} - 1} \right){\text{B}}\,{\text{dt}}^{{\prime }} ,} & {{\text{L}}^{{\prime }} > 0} \\ {{\text{k}}^{{\prime }}_{{{\text{sedimentation}}}} \left( {1 - {\text{L}}^{{\prime }} } \right){\text{B}}\,{\text{dt}}^{{\prime }} ,} & {{\text{L}}^{{\prime }} < 0} \\ \end{array} } \right.$$

where ${\text{k}}^{{\prime }}$ is the erosion/sedimentation rate, and ${\text{dt}}^{{\prime }}$ is the time increment. Equations 4.1–4.8 are solved implicitly by relying on the PETSc-SNES class objects^75^.

### Supplementary Information

Supplementary Information.

## Data Availability

The data used can be found in the corresponding cited references. The software used for the temperature calculation, GOLEM can be found at https://doi.org/10.5281/zenodo.999401. The code used for converting seismic velocities to temperatures can be downloaded from https://doi.org/10.5281/zenodo.6538257 or https://github.com/ajay6763/V2RhoT_gibbs.git. The code for the stability analysis can be found at https://github.com/mcacace/pelycan. Post-processing and plotting of the results were done using GMT6^78^, Matplotlib^81^, and Inkscape (https://inkscape.org/). Scientific colour maps from^82^ were used for visualization.
