# Supplementary material for: Thermodynamics of continental deformation
Source: Sci Rep. 2023 Nov 14;13:19920. doi: 10.1038/s41598-023-47054-3 (PMC10646047; doi:10.1038/s41598-023-47054-3)
Supplement: Supplementary file 1 — Supplementary Information. [file 41598_2023_47054_MOESM1_ESM.pdf]

# Supplementary material
# Thermodynamics of continental deformation

**Ajay Kumar[1*], Mauro Cacace[1], Magdalena Scheck-Wenderoth[1,2]**

[1]GFZ German Research Centre for Geosciences, Potsdam Germany
[2]Faculty of Georesources and Materials Engineering, RWTH Aachen University, Aachen, Germany.
*kumar@gfz-potsdam.de, ajay6763@gmail.com

**This file contains: Table S1 and Figures S1-S11**

**Table S1 | Rheological properties of the additionally tested lithologies.**

| Lithology | Power-law strain rate A ($Pa^{-n}\ s^{-1}$) | Power-law Exponent n | Activation Enthalpy H ($J\ mol^{-1}$) |
|---|---|---|---|
| **Quartzite wet** (Ranalli and Murphy 1987) | 3.99 E-18 | 2.3 | 1.54E+05 |
| **Quartzite dry** (Ranalli and Murphy 1987) | 2.51 E-20 | 2.4 | 1.56E+05 |
| **Peridotite wet** (Hirth and Kohlstedt 1996) | 5.012 E-15 | 3.5 | 5.15E+05 |

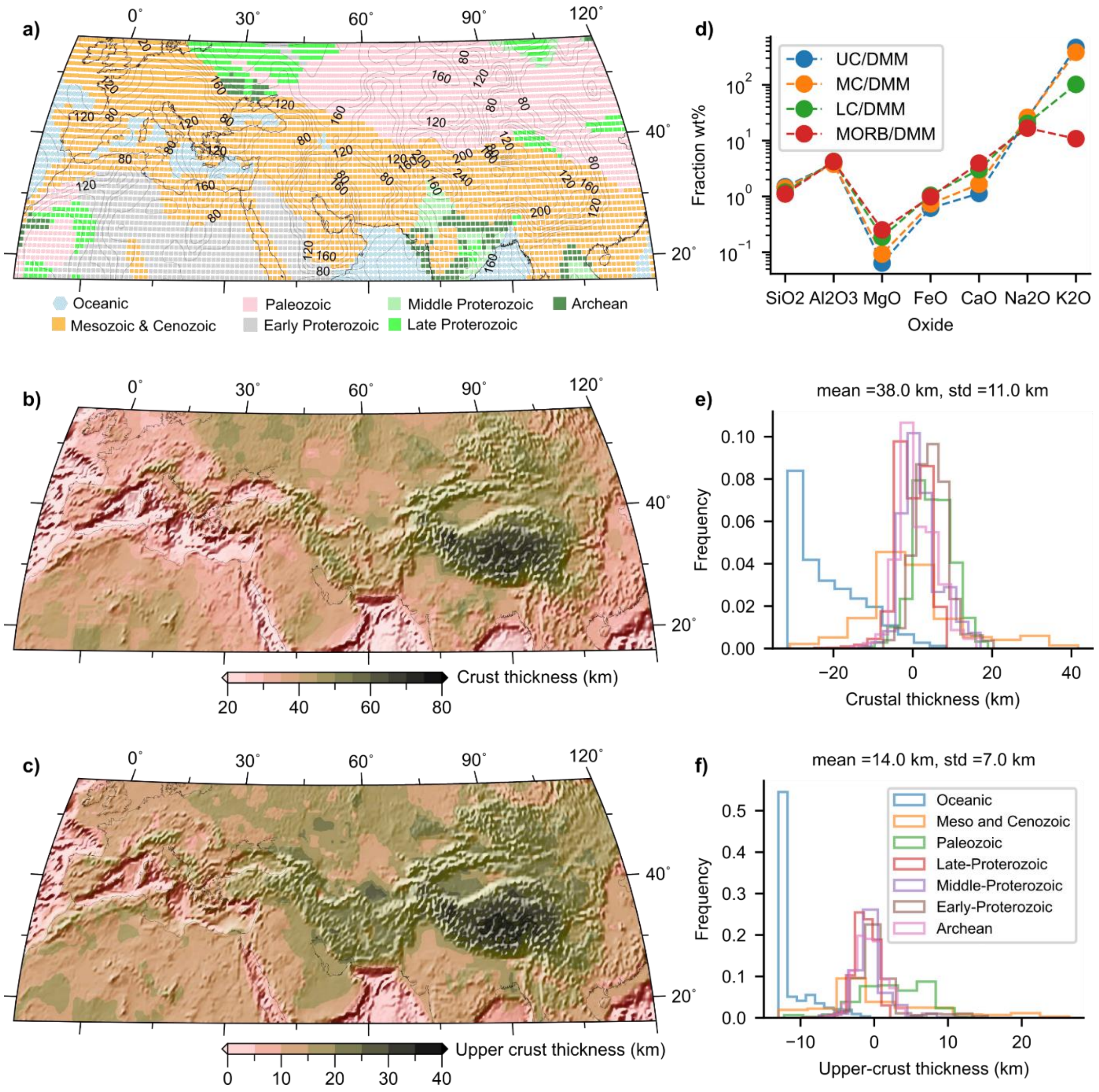


**Fig. S1 | The Alpine Himalayan Collision Zone**. a) Map of the Alpine Himalayan Collision Zone (AHCZ) depicting the different tectonothermal ages (Goutorbe et al. 2011). Grey contours mark the lithosphere-asthenosphere boundary (LAB) depth (Priestley, McKenzie, and Ho 2018). All major orogens (Tibet, Zagros) have a thicker lithosphere as that found beneath stable intracontinental regions. b) and c) Thickness of the crust and upper-crust in the AHCZ, respectively (Laske et al. 2013). Note the decreasing trend, from the east to the west, of crustal thickness in (b) beneath orogens. The upper-crust is thicker beneath the orogens compared to the stable continental interior regions. d) Major oxides ratio of continental crustal layers and mid-oceanic-ridge-basalt (MORB) (Rudnick and Gao 2003) normalized with upper-mantle composition (DMM (Workman and Hart 2005)). Note the enrichment of radioactive $K_2O$ in the crust with respect to the MORB. e) Distribution of total crustal thickness, also shown in terms of deviations from its mean value. In the Mesozoic-Cenozoic regions, crustal thickness show both thickening, almost double (e.g., in Tibet (b), and thinning (orange curve). f) Distribution of upper-crust thickness as a function of its tectonothermal age, shown as deviation from its mean value in the AHCZ. In the Mesozoic-Cenozoic regions, the upper-crust is anomalously thick (orange curve). Maps were made using Generic Mapping Tools Version 6 (Wessel et al. 2019).

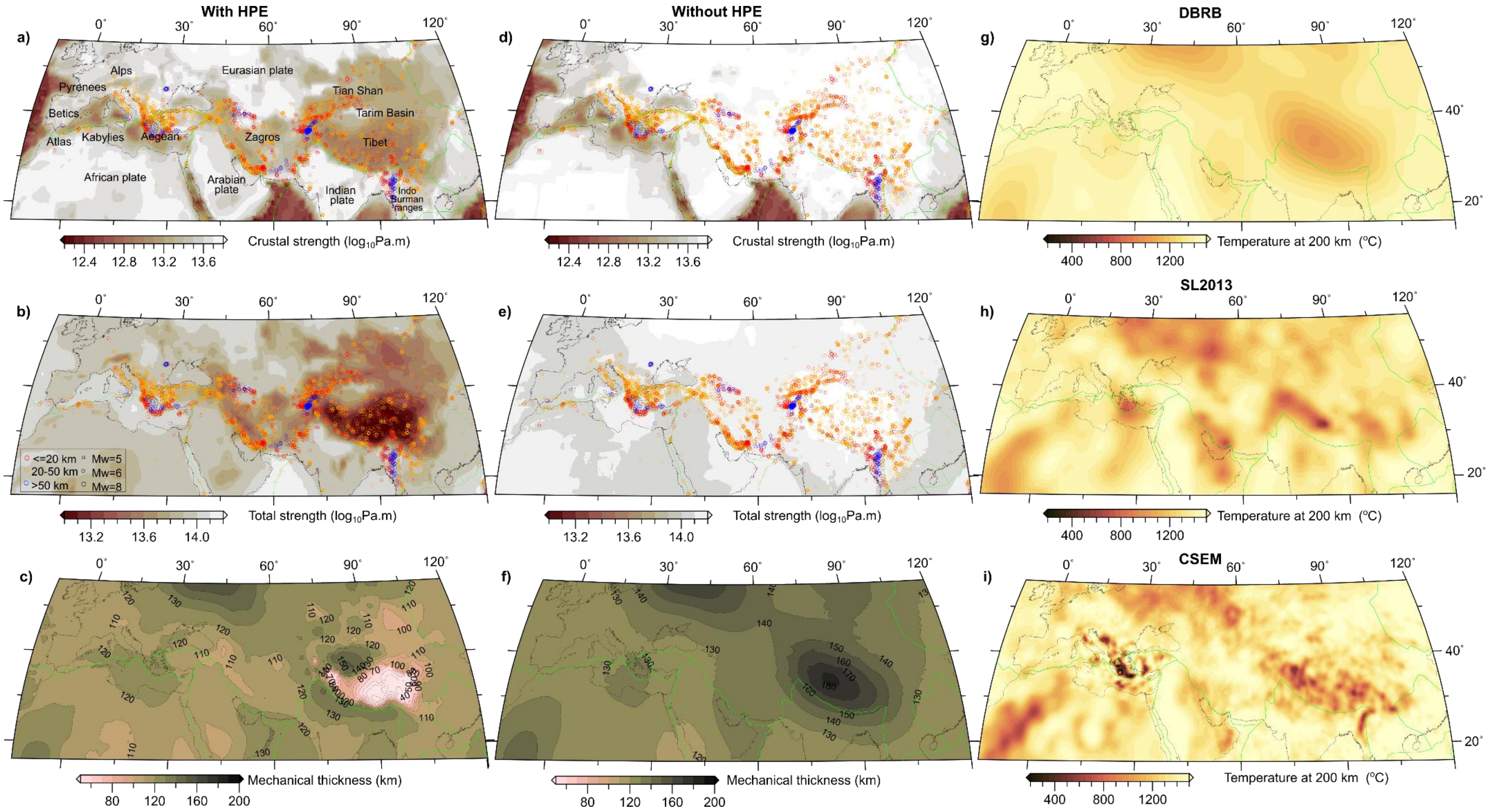


**Fig. S2 | Effect of heat producing elements (HPE) on the long-term strength and mechanical thickness of the AHCZ**. a) Integrated strength of the crust (integrated up to the Moho depth), b) integrated strength of the lithosphere (integrated up to the base of the model), c) Mechanical thickness of the lithosphere, for the model in the main text (Fig.1). Mechanical thickness is defined as the depth up to which strength is above 10 MPa (Ranalli 1994), thus representing effective thickness of the stress bearing portion of the plate. Similarly, in d-f) where all parameters are the same as in a-c), except the effect of HPE is neglected by setting radiogenic heat production to zero in the model. g) Temperature boundary condition at the base of the model (200 km) used in a-f), derived from the conversion of the shear-wave velocities from DBRB tomography model (Debayle et al. 2020) to temperature (see Methods). Neglecting the RHP increases the strength of the crust (d), and lithosphere (e) in the orogens, and reflects the temperatures at the base of the model (g), such that hotter domains (e.g., Red Sea Rift, Western Europe) are relatively weaker and show thinner mechanical thickness than colder domains (e.g., orogens, stable continental interiors) that have a higher mechanical thickness (f). h) and i) show temperatures at 200 km depth derived from the SL2013 (Schaeffer and Lebedev 2013) and CSEM (Fichtner et al. 2013) tomography models, respectively (see Fig. S4). Maps were made using Generic Mapping Tools Version 6 (Wessel et al. 2019).

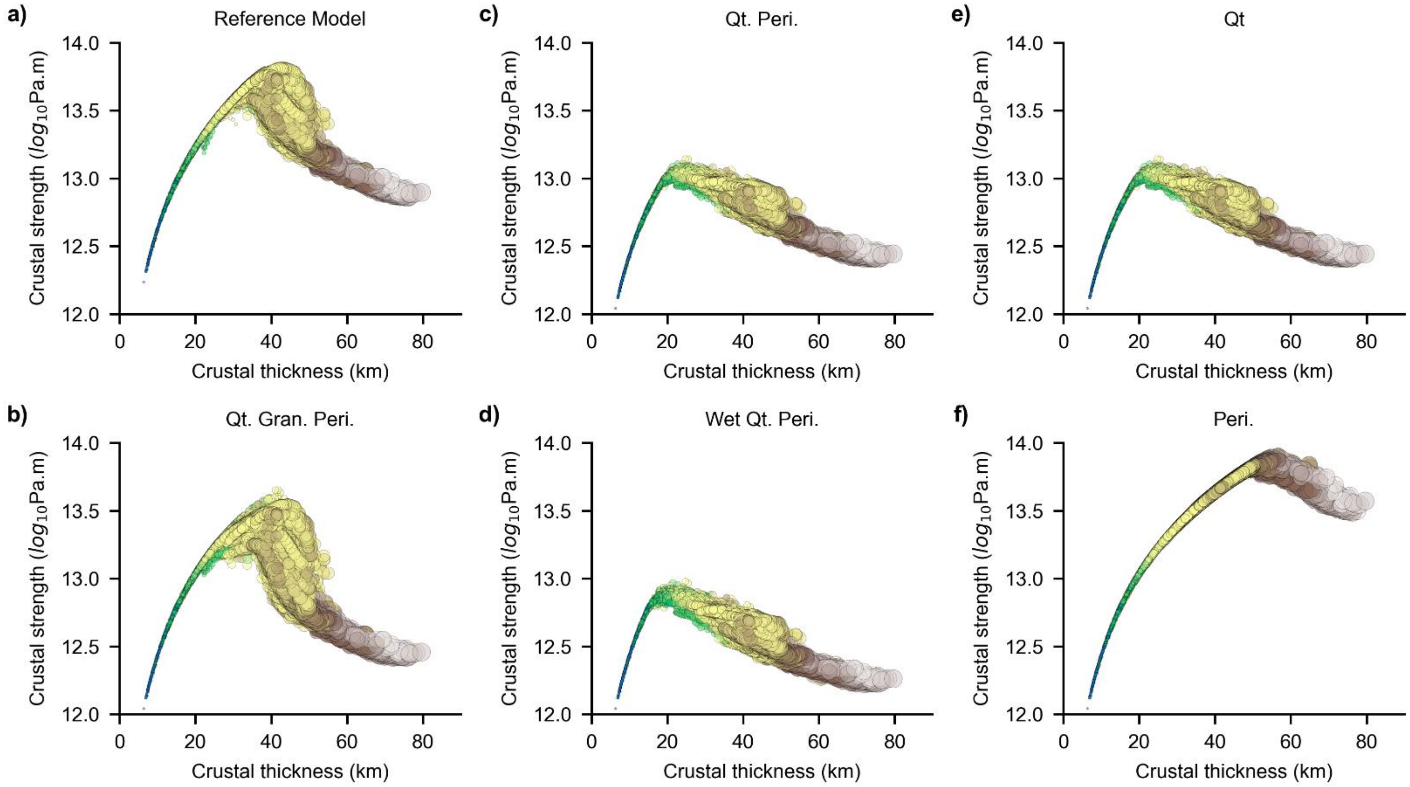


**Fig. S3 | Effect of composition and rheological parameters on the critical crustal thickness.** a) Crustal strength versus crustal thickness corresponding to the reference model (Fig. 1 and Table 1 in the main text). Additionally, tested compositions are listed in Table S1. In b) Dry quartzite (Ranalli and Murphy 1987) composition in the sediments, upper-crust and middle-crust, mafic-granulite (Wilks and Carter 1990) composition in lower-crust and dry peridotite (Hirth and Kohlstedt 1996) composition in the mantle. In c) dry quartzite composition in all the crustal layers and dry peridotite in the mantle. d) Same as in c) but with wet compositions. In e) and f) dry quartzite and peridotite compositions are used in the entire model, respectively. Wet compositions decrease the strength of the crust without drastically changing the critical crustal thickness (c and d). Whereas, the critical crustal thickness increases with the mafic composition (f) and decreases with the felsic composition (e).

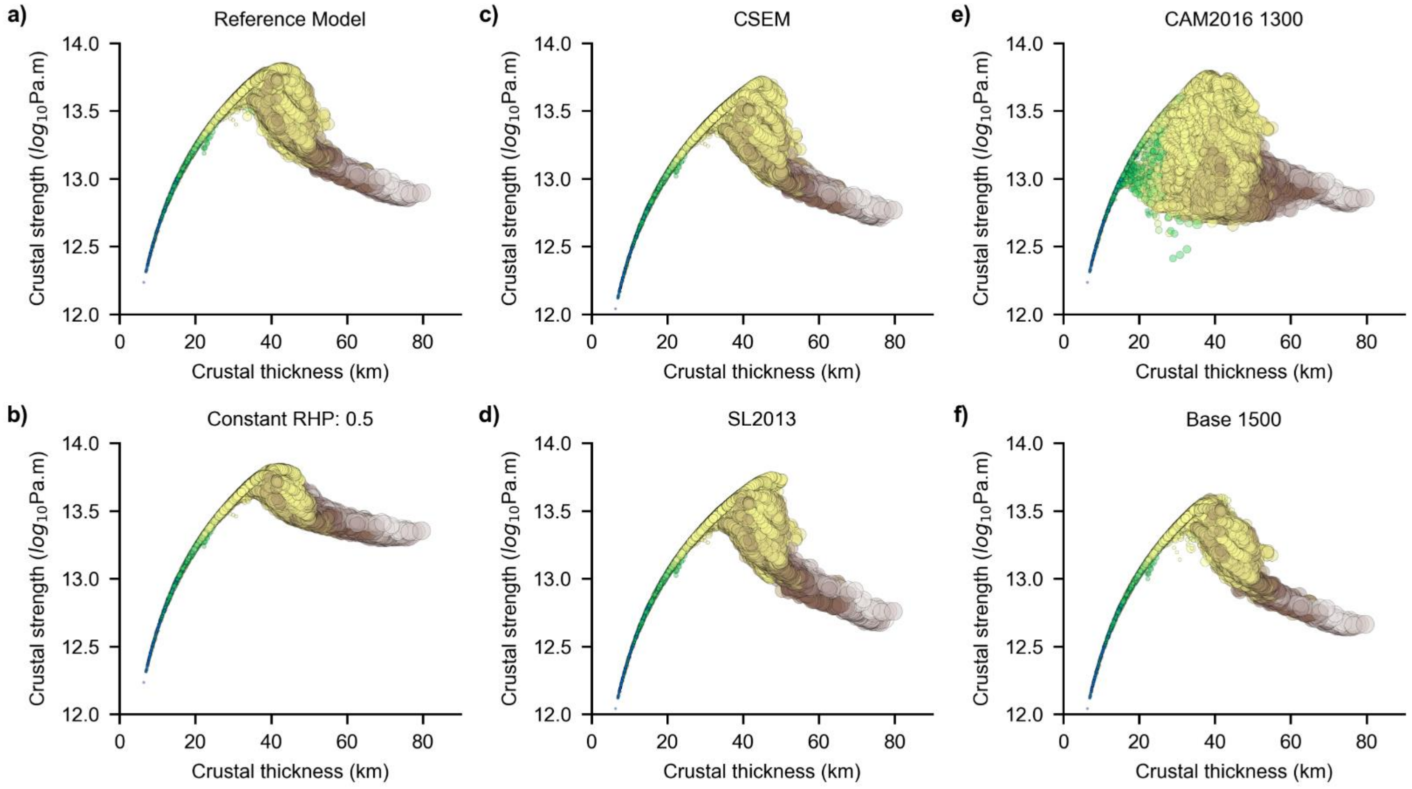


**Fig. S4 | Effect of mantle thermal boundary condition on the critical crustal thickness and strength**. a) Crustal strength versus crustal thickness for tectonothermal lithospheres corresponding to the reference model (Fig. 1c in the main text, Table S1). In b) constant and lower than average (compared to a) RHP of 0.5 E-06 W/m$^3$ is used in all the crustal layers, which leads to less scatter around the critical crustal thickness and a slightly stronger crust above the critical crustal thickness, highlighting the role of RHP in weakening (a and b). In c) and d) temperatures at 200 km depth are from the conversion ( see Fig. S2h&i and Methods) of CSEM (Fichtner et al. 2013) and SL2013 (Schaeffer and Lebedev 2013) tomography models, respectively. In e) the base of the model is set to the LAB depths from the CAM2016 model (Priestley et al. 2018) and the temperature is set to 1300 °C. In f) temperature at the base of the model (200 km) is set to 1500 °C. To first order, the temperature boundary condition at 200 km does not change the critical crustal thickness drastically (c-f). Different tomography models (c-d) do not change the first-order crustal thickness behaviour, rather they produce slightly different spread in crustal strength above critical crustal thickness.

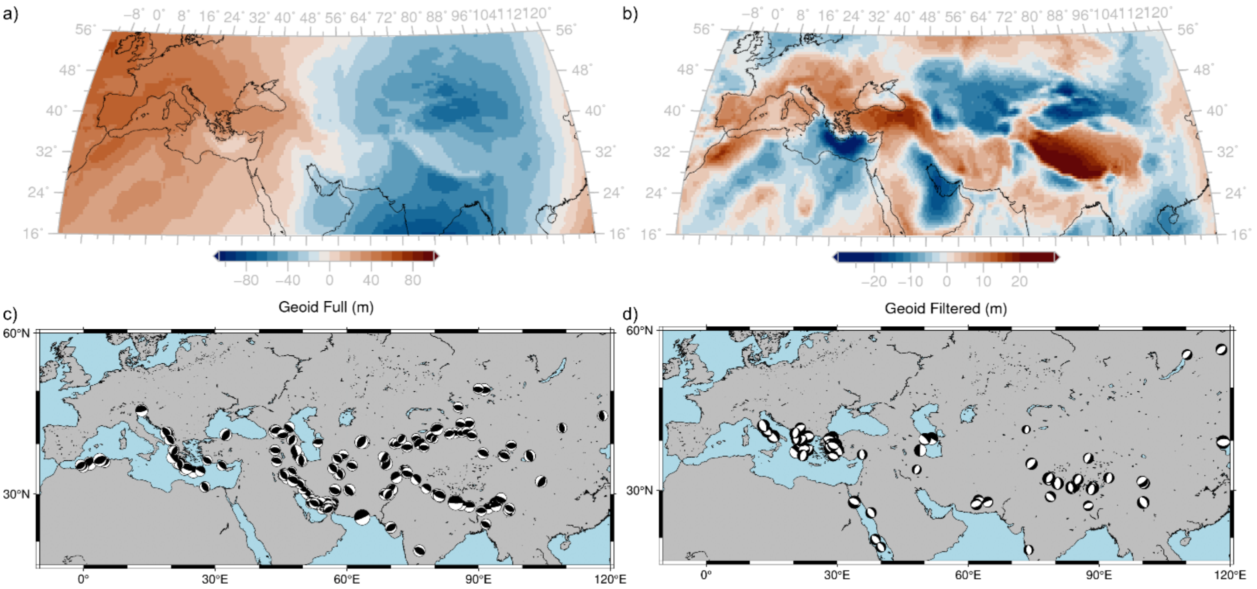


**Fig. S5 | Map of the geoid height anomaly and earthquake fault plane solutions in the Alpine Himalayan Collision zone.** a) Full geoid height anomaly in the AHCZ from ICGEM using the GECO model (Sinem Ince et al. 2019). b) Geoid height anomaly filtered to a degree and order 10. Note the positive geoid height anomalies in the major orogens in AHCZ (b) compared to long-wavelength positive and negative anomalies in a). In c) and d) seismic waveform modelling constrained depth ($\leq$ 80 km) and fault-plane solutions of earthquakes taken from the compilation in gWFM_v_1.2 (Wimpenny and Watson 2021) are plotted. In c) thrust earthquakes and in d) normal fault earthquakes are plotted. Maps were made using Generic Mapping Tools Version 6 (Wessel et al. 2019).

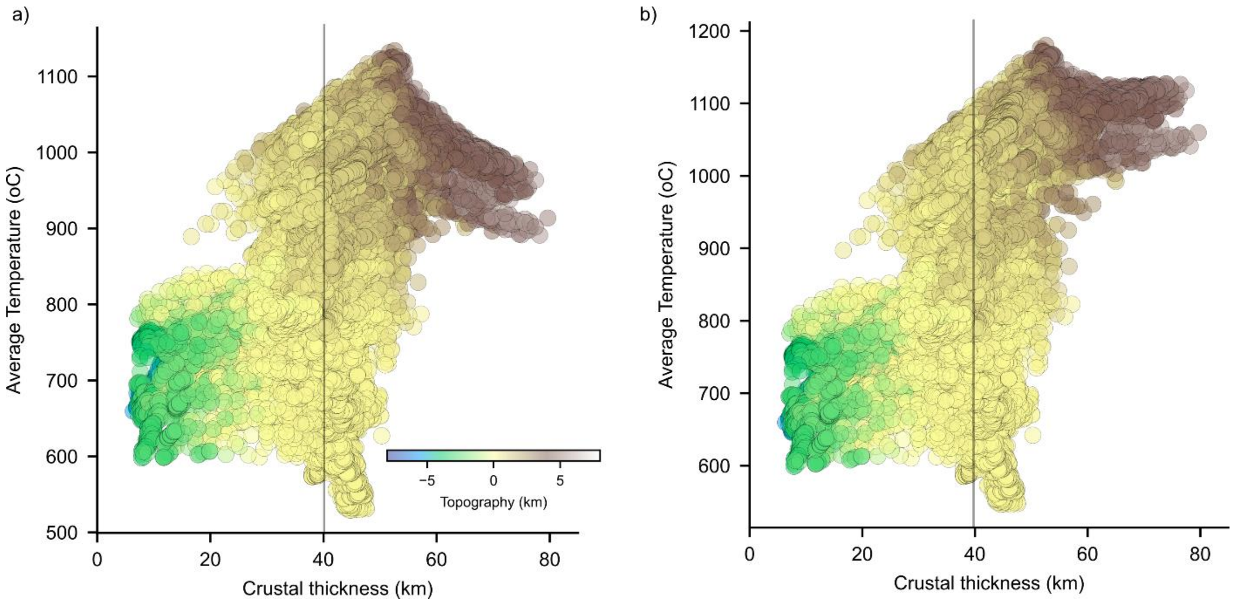


**Fig. S6 | Average temperature of the lithosphere versus crustal thickness in the AHCZ.** In order to derive temperature in the mantle from 50 km to 150km depth, shear-wave velocities from the DBRB tomography model (Debayle et al. 2020) are converted to temperature (See Section 2 in Methods). From 50 km depth to the surface temperatures are derived by solving the steady-state temperature equation where bottom boundary condition is taken from the tomography derived temperatures (See Section 2 in Methods). In a) HPE in top 50 km depending on the crustal thickness are not considered whereas in b) HPE are considered (Table 1). Because of the HPE, temperatures are almost constant in the thick crust regions. Regions with topography close to the global average (~ 100m) are colder and show crustal thickness similar to the equilibrium critical crustal thickness. Top right corner in both figures resembles the phase space presented for the orogen style perturbations (Fig. 5).

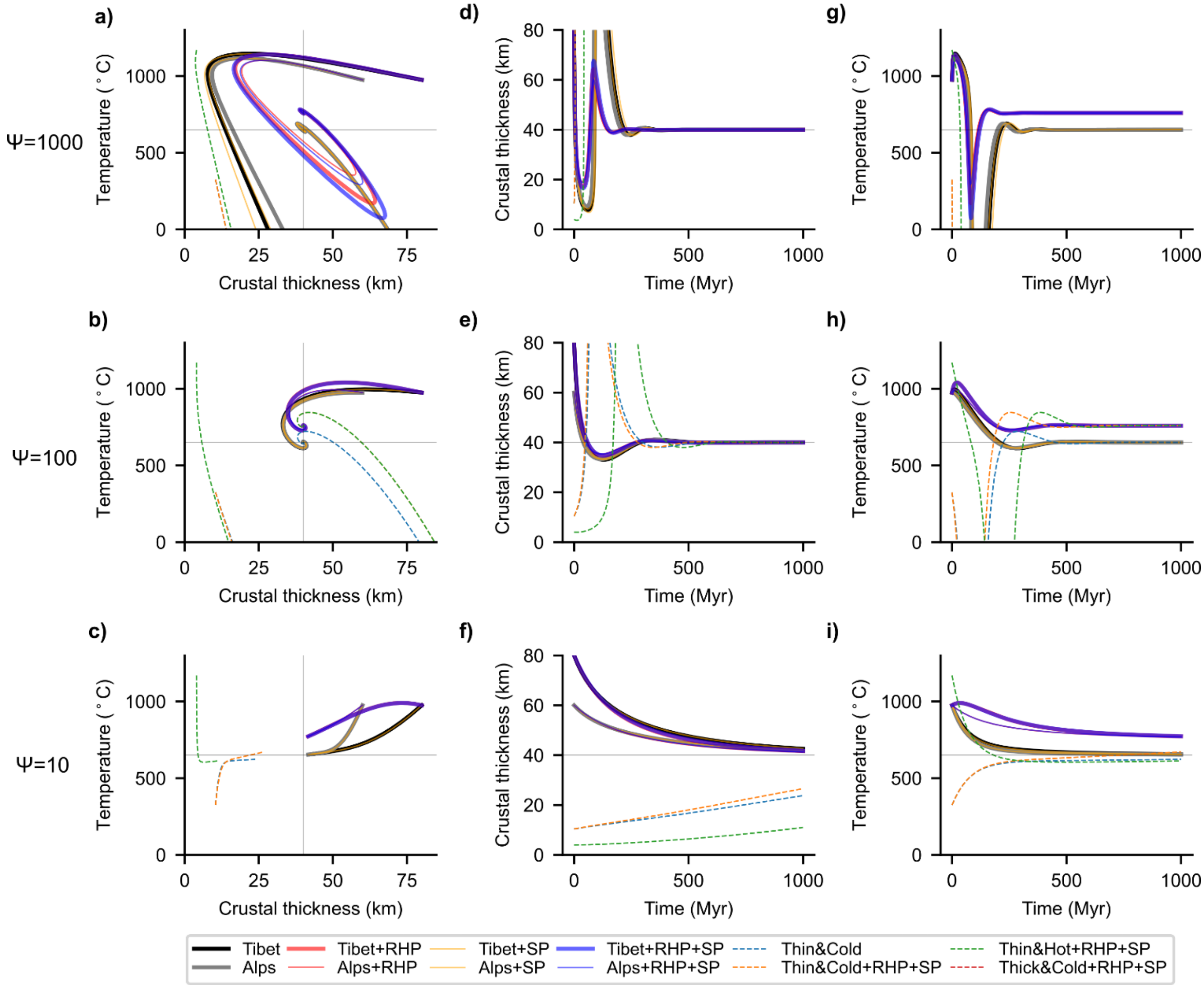


**Fig. S7 | Stability and evolution of lithosphere for temperature independent rheology.** Energy parameter, $\psi$, which is the ratio of the thermal and mechanical relaxation time scales, increases from bottom to top. Each row represents an experiment with a fixed $\psi$, where panels: a-c) show the phase-diagram, d-f) time evolution of thickness, g-i) time evolution of temperature. Oscillations in the crustal thickness and temperature evolution increase with $\psi$, leading to run-away instability for $\psi \geq 1000$ (top row).

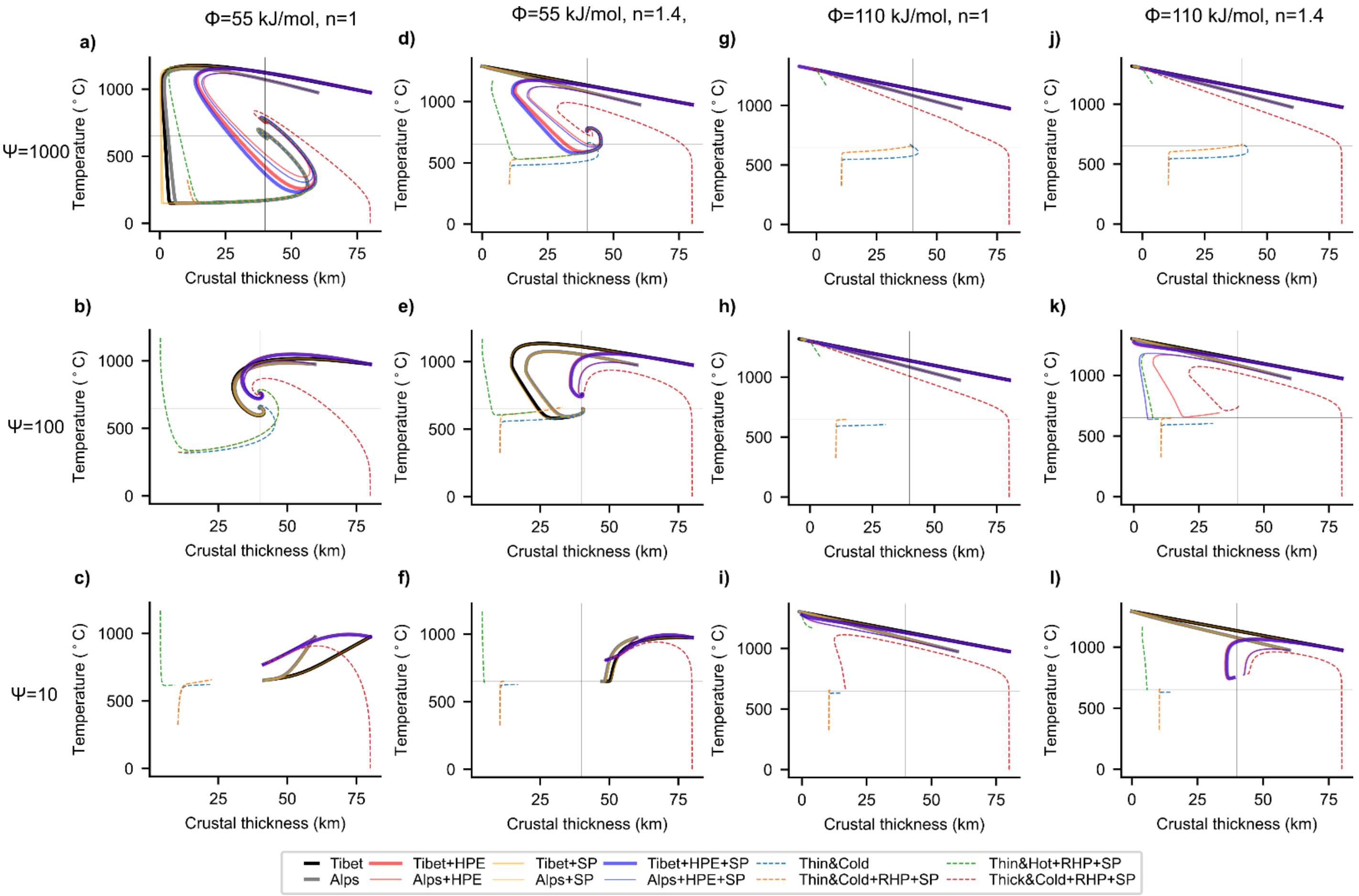


**Fig. S8 | Stability and evolution of lithosphere for temperature dependent and non-linear rheology.** Each row represents an experiment with different energy parameters, ψ, and each column with different creep exponent, n, and creep activation energy, ϕ. Oscillations in the crustal thickness and temperature evolution are limited by the creep activation energy and exponent. Activation energy for the creep (i.e., temperature dependence) limit the oscillations such that the run-away extension without it at ψ = 1000 (Fig. S7 left column) are attracted towards the equilibrium (see left column: a-c). On increasing the activation creep energy, oscillations are additionally limited by the non-linear rheology, n = 1.4 (rows: left to right), to damp the exponential growing oscillations.

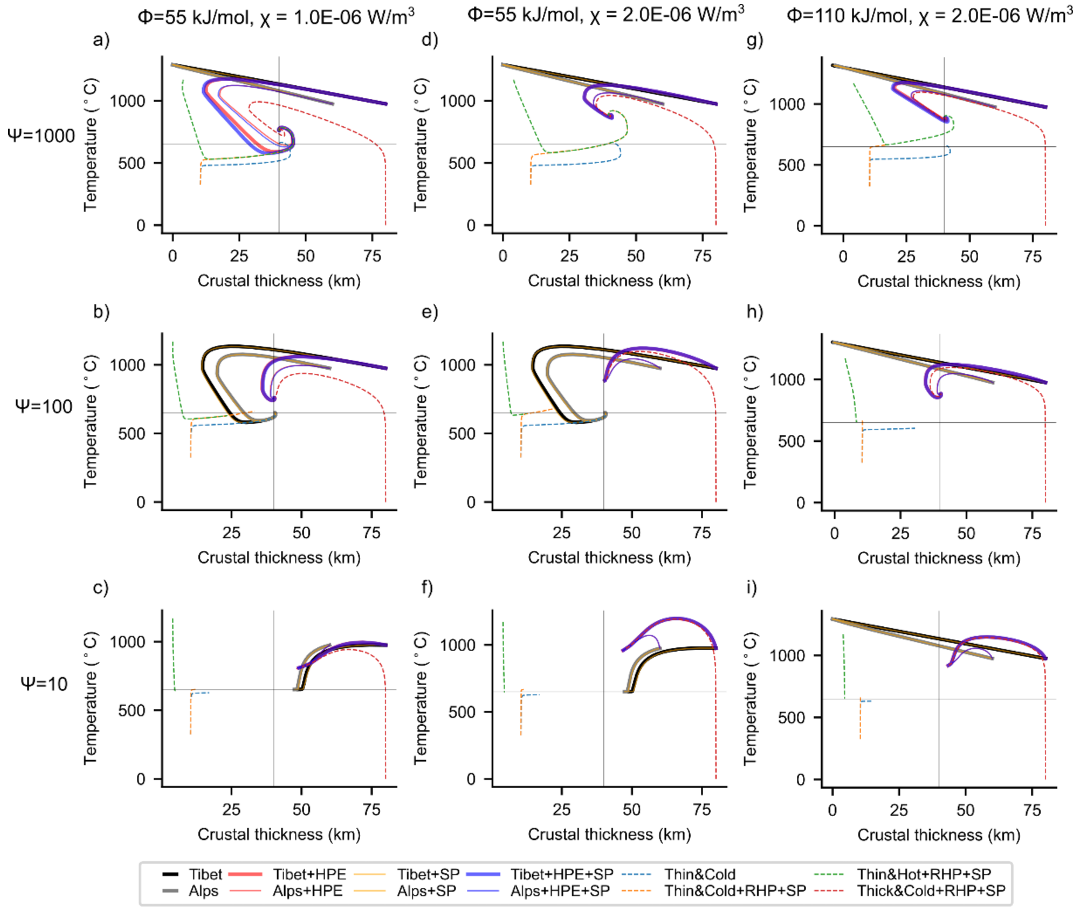


**Fig. S9 | Energy dependence on the phase space.** Each row represents an experiment with different ψ (see the row title), and each column with different creep activation energy, ϕ, RHP, χ (see the column title) and n =1.4. Each panel show the evolution of the perturbations in the phase space. Decreasing the energy parameter (top to bottom), increases the overall viscosity, leading to longer time required for the perturbations to come back to the equilibrium. Increasing the RHP and activation energy (left to right) dampens the oscillation in crustal thickness leading to relatively shorter time to come back to the equilibrium.

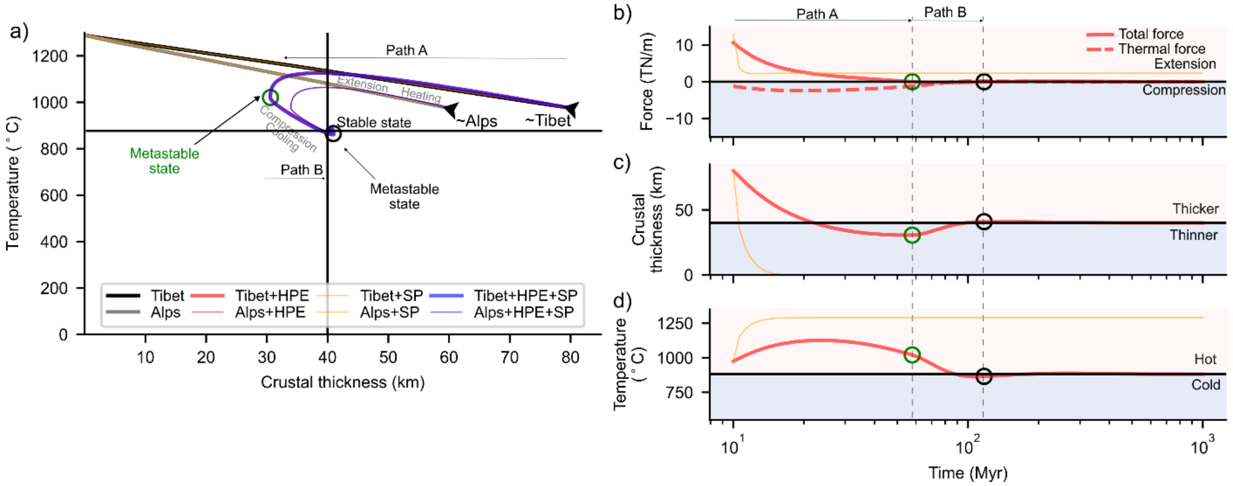


**Fig. S10 | Stability and evolution of the orogenic lithosphere with enhanced HPE.** In these experiments, all the parameters are the same as in Figure 5 in the main text except that the HPE are doubled.

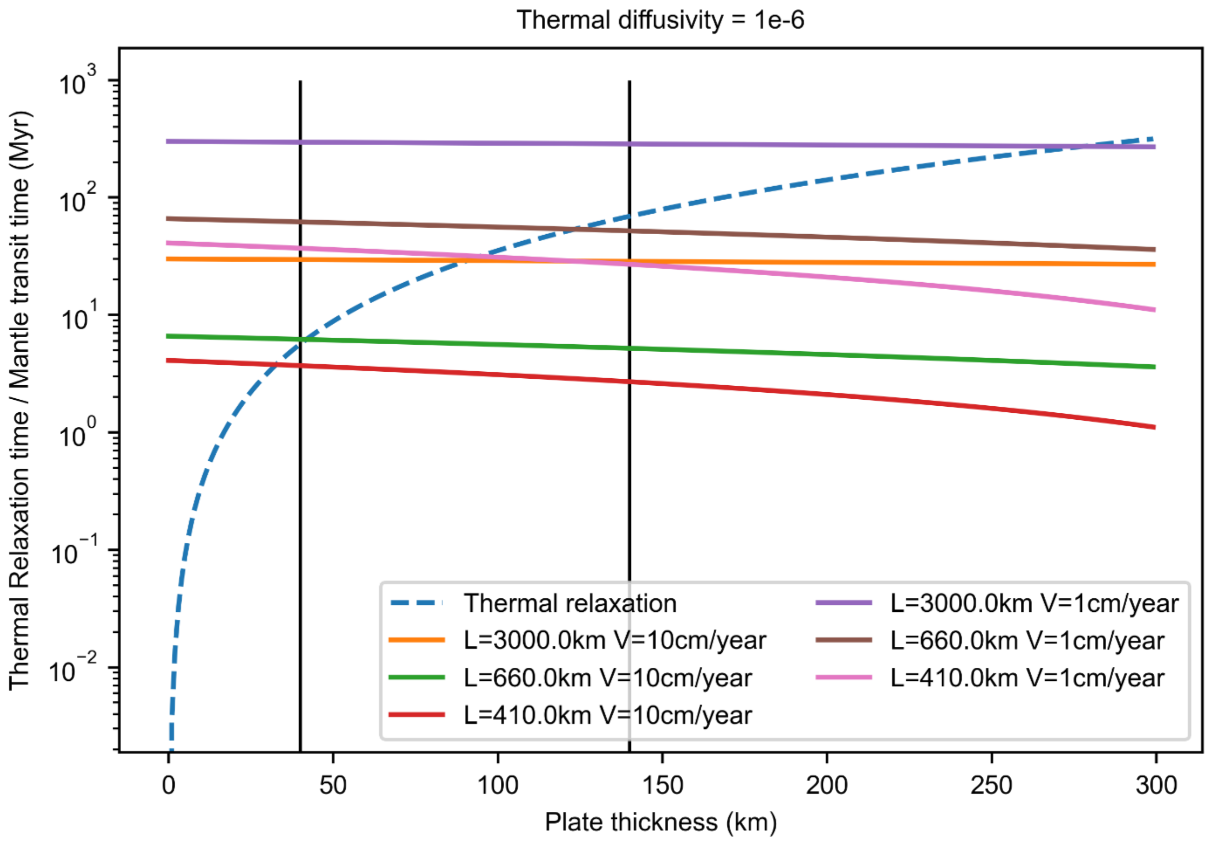


**Fig. S11 | Log-log plot of the time constants for the thermal relaxation and mantle convection.** Thermal relaxation time (dashed curve) is computed using $\tau_{Thermal} \sim S^2/\pi^2 K$ where S is the thickness of the plate, and K is the thermal diffusivity equal to 1E-06 $m^2/s$. Mantle convection transit time (solid curves) is calculated using $\tau_{Convection} \sim (D-S)/V$ where D-S is the length over which convection takes place, D is the depth at which convection starts, coloured solid lines (for 3000 km, 660 km, and 410 km), and S is the thickness of the plate (along the x-axis), and V is characteristic flow velocity with end-member velocities of 1cm/yr and 10 cm/yr (Ogawa 2008; Tackley 2000). Solid vertical lines denote a plate crustal thickness of 40 km and a plate thickness of 140 km.
